# The complete mitochondrial genome of *Chionodraco rastrospinosus* (Notothenioidei: Channichthyidae) with phylogenetic consideration

**DOI:** 10.1080/23802359.2018.1483769

**Published:** 2018-07-27

**Authors:** Shuzhang Liang, Wei Song, Hongliang Huang, Taichun Qu, Fengying Zhang, Keji Jiang, Xuezhong Chen, Lingbo Ma

**Affiliations:** a Key Laboratory of Oceanic and Polar Fisheries, Ministry of Agriculture, East China Sea Fisheries Research Institute, Chinese Academy of Fishery Sciences, Shanghai, China;; b College of Fisheries and Life Sciences, Shanghai Ocean University, Shanghai, China

**Keywords:** Chionodraco rastrospinosus, mito-genome, genome structure, phylogenetic tree

## Abstract

In this study, the complete mitochondrial genome of *Chionodraco rastrospinosus* was obtained, which was 17598 bp including 2 ribosomal RNAs, 13 protein-coding genes, 22 transfer RNAs, and a non-coding control region. The length of D-loop was 1332 bp and its contents of A, T, C, and G were 30.3%, 27.6%, 26.8%, and 15.3%. The complete mtDNA sequences of *C. rastrospinosus* and other 14 species were used to reconstruct the phylogenetic tree, suggested that *C. rastrospinosus* was closest to two species of *Chionodraco.* The study would provide a basic data for further research on population structure, conservation genetics and molecular evolution of *C. rastrospinosus*.

*Chionodraco rastrospinosus* is widely distributed in the South Shetland Islands, the South Orkney Islands, and the Antarctic Peninsula (Kock K-H 1993). In these areas, it is found most commonly at depths from 200–400 m where the temperature is close to –1 °C. Because of genetic mutations, they completely lack the oxygen-binding haemoglobin and the colour of blood is translucent instead of red (Kock K-H 2005). Papetti reported the partial sequence of *C. rastrospinosus* and no *ND6* gene (Papetti et al. 2007). However, the current study found that *Chionodraco hamatus* (Song et al. 2016), which also belonged to *Chionodraco,* had *ND6* gene, so we did the research. Besides, the complete mitochondrial genome sequence of *C. rastrospinosus* would be useful for understanding population structure, molecular systematic, and conservation genetics.

Adult fish of *C. rastrospinosus* was collected from Antarctic (62°52′54″S, 59°28′54″W), it was transported to East China Sea Fisheries Research Institute Chinese Academy of Fishery Science after freezing at –80 °C. The genomic DNA was extracted from muscle tissues and sequenced using the Illumina HiSeq2000 platform (Illumina, San Diego, CA). The primers for amplification were designed according to the sequence of *C. hamatus* (KT921282). We obtained the complete mitogenome and submitted it to Genbank database with an accession number MF622064.

It was a circular molecule of 17598 bp in length, including 2 rRNAs, 13 protein-coding genes, 22 tRNAs, and a non-coding control region. The contents of A, T, C, and G were 26.42%, 26.10%, 30.05%, and 17.41%, respectively. Among the 22 tRNAs, 8 tRNAs (tRNA^Pro^, tRNA^Glu^, tRNA^Ser^, tRNA^Tyr^, tRNA^Cys^, tRNA^Asn^, tRNA^Ala^, and tRNA^Gln^) were encoded by L-strand, when others were encoded by H-strand. In 13 protein-coding genes, two kinds of start codons were identified, two (CO1 and ATP6) were started with GTG, whereas others started with ATG. Five genes (ND1, CO1, ATP8, ATP6, and ND4L) ended with TAA or TAG and the others ended with T or TA. The length of D-loop was 1332 bp, it was shorter than *C. hamatus* and its overall base composition was 30.3% for A, 27.6% for T, 26.8% for C, and 15.3% for G, with a high A + T content of 57.9%, accord with the structural characteristics of AT rich.

Phylogenetic analysis was performed using the Neighbour-joining method in MEGA 5.0 based on complete mitogenome of *C. rastrospinosus* and other 14 species (Figure 1). The phylogenetic tree showed that *C. rastrospinosus* clustered with *C. hamatus* and *C. myersi* and then together with other two fishes in family Channichthyidae. The result was similar to the outcome of Song’s research on *C. hamatus* (Song et al. 2016).

**Figure 1.**
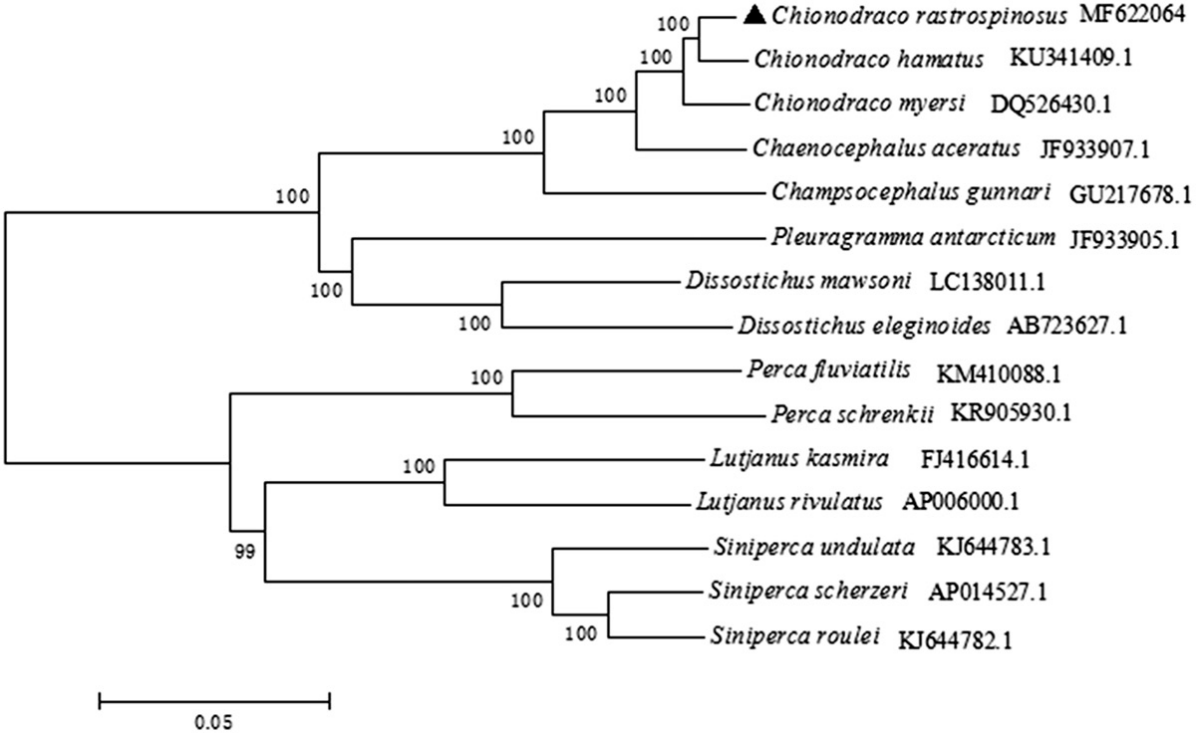
The phylogenetic tree based on complete mtDNA sequences using the neighbour-joining method in MEGA 5.1. *C. rastrospinosus* was highlighted with a black triangle.
